# Temperature Dependence of Deformation Behaviors in High Manganese Austenitic Steel for Cryogenic Applications

**DOI:** 10.3390/ma14185426

**Published:** 2021-09-19

**Authors:** Jun Chen, Shuang Li, Jia-Kuan Ren, Zhen-Yu Liu

**Affiliations:** State Key Laboratory of Rolling and Automation, Northeastern University, Shenyang 110819, China; neuwow@163.com (S.L.); 18233561905@163.com (J.-K.R.); zyliu@mail.neu.edu.cn (Z.-Y.L.)

**Keywords:** high manganese austenitic steel, strength and ductility, mechanical twinning, dislocation structure

## Abstract

The deformation structure and its contribution to strain hardening of a high manganese austenitic steel were investigated after tensile deformation at 298 K, 77 K and 4 K by means of electron backscatter diffraction and transmission electron microscopy, exhibiting a strong dependence of strain hardening and deformation structure on deformation temperature. It was demonstrated that sufficient twinning indeed provides a high and stable strain hardening capacity, leading to a simultaneous increase in strength and ductility at 77 K compared with the tensile deformation at 298 K. Moreover, although the SFE of the steel is ~34.4 mJ/m^2^ at 4 K, sufficient twinning was not observed, indicating that the mechanical twinning is hard to activate at 4 K. However, numerous planar dislocation arrays and microbands can be observed, and these substructures may be a reason for multi-peak strain hardening behaviors at 4 K. They can also provide certain strain hardening capacity, and a relatively high total elongation of ~48% can be obtained at 4 K. In addition, it was found that the yield strength (YS) and ultimate tensile strength (UTS) linearly increases with the lowering of the deformation temperature from 298 K to 4 K, and the increment in YS and UTS was estimated to be 2.13 and 2.43 MPa per 1 K reduction, respectively.

## 1. Introduction

For a long time, high manganese austenitic steels, such as high manganese twinning-induced plasticity (TWIP), transformation-induced plasticity (TRIP) and low-density steels, have attracted much interest due to their potential applications in the automotive industry [1,2,3,4,5]. A large number of studies focus on deformation mechanisms, strain hardening, yield strength, texture, fracture and fatigue, etc. Up to now, these steels have not been widely used in the automotive industry because of their relatively high cost compared with conventional automobile steels, hydrogen-induced cracks and other problems. Recently, high manganese austenitic steels were shown to act as a candidate cryogenic material for liquefied natural gas (LNG) transportation by ship and truck due to their extraordinary cryogenic mechanical properties and relatively low cost compared with conventional cryogenic materials [6,7,8]. Moreover, tensile and impact properties at room temperature and 77 K, as well as corresponding deformation mechanisms, were investigated in detail [6,7,8,9,10,11,12], indicating that these steels have potential applications in the LNG field, and they have been used in LNG tank building. However, there are few data on plastic properties of high manganese austenitic steels at a temperature as low as 4 K. These extremely cryogenic plastic properties determine whether they can be used in extremely cryogenic fields, such as liquid hydrogen and liquid helium.

In addition, the mechanical twinning, as one of the secondary plastic deformation mechanisms [1,2,13], plays a significant role in the enhancement of strain hardening and the improvement of ductility. Recently, Luo et al. [14] reported that high manganese TWIP steels can also achieve high strain-hardening rates without mechanical twining at 373 K and 473 K. However, it is commonly accepted that the combination of strength and ductility in high manganese TWIP steel results from the high strain-hardening rates caused by mechanical twinning [1,2,15,16]. The stacking fault energy (SFE) increases with increasing temperature [17], leading to the suppression of mechanical twinning at high temperatures. Thus, at low temperatures of 77 K and 4 K, the SFE may be less than 15~20 mJ/m^2^. The TRIP effect can occur, and extremely low temperature mechanical properties are deteriorated. Hence, we designed a Fe–0.6C–0.5Si–24.2Mn–4.9Al (in wt%) steel with a relatively high SFE at room temperature to suppress martensite transformation at 77 K and 4 K. In addition, the deformation mechanisms are somewhat unclear at 4 K. Moreover, it is also unknown whether sufficient twinning can occur for a twinning SFE at 4 K.

Hence, we investigate the temperature dependence of deformation behaviors, mainly focusing on mechanical twinning and planar dislocation slipping. The present study will provide a better understanding of the role of mechanical twining in the enhancement of the strain hardening rate and deformation mechanism at a temperature as low as 4 K.

## 2. Experimental Procedures

### 2.1. Material Preparation

The high manganese steel was alloyed by ~4.9 wt% Al to increase the SFE, with the aim of suppressing martensite transformation at 4 K. The chemical compositions and SFEs, which were estimated via a thermodynamic model [17], are given in Table 1. The steel was melted in a high-frequency vacuum induction furnace with an argon protective atmosphere and cast into an iron mold. After that, the ingot was air cooled to room temperature with a cooling rate of ~20 °C/min. The ingot was hot-rolled to a thickness of ~12 mm at a temperature range of 1150–1050 °C and subsequently water-quenched to room temperature. After that, the hot-rolled plate was isothermally treated at 1200 °C for 2 h to eliminate segregation.

### 2.2. Tensile Test

Standard cylindrical tensile samples with a gauge diameter of 6 mm and a gauge length of 50 mm were machined along the rolling direction. The uniaxial tensile testing was performed on an AG-X plus pc-controlled tensile machine (Shimadzu Co. Ltd., Kyoto, Japan) at 298 and 77 K at a cross speed of 5 mm/min. The 4 K tensile testing was performed on an MTS-SANS CMT5000 pc-controlled tensile machine (MTS SYSTEMS (CHINA) Co. Ltd., Shanghai, China) equipped with a CryoLab cryogenic system (4.2~300 K) (Self-research system by Technical Institute of Physics and Chemistry, CAS, Beijing, China) at a cross speed of 5 mm/min. Moreover, the steels with tensile deformation at 298, 77 and 4 K were designated as the 298 K, 77 K and 4 K steels, respectively.

### 2.3. Microstructure Characterizations

#### 2.3.1. Electron Backscatter Diffraction

Metallographic specimens were electron-discharge machined from the hot rolled steel and tensile fractured samples with a maximum uniform strain. These specimens were mechanically polished using silicon papers and a polishing machine with the help of polishing paste. Subsequently, they were further polished using a three ion beam polishing instrument (Leica EM TIC 3X, Leica Microsystems Ltd., Wetzlar, Germany) to remove the surface strain layers. The microstructure characteristics before and after tensile deformation were analyzed using a Zeiss Ultra 55 (Carl Zeiss AG, Jena, Germany) field-emission scanning electron microscope (FE-SEM) equipped with an Electron backscatter diffraction (EBSD) attachment. Moreover, a scanning step of 5 μm, which is small enough to obtain a clear morphology, was utilized during EBSD data collection. The analyzed area of ~2.3 × ~1.7 mm was sufficiently large. These EBSD data were post-processed by a Tango procedure using HKL CHANNEL 5 software (version; 5.0.9.0, OXIG Co. Ltd., Oxford, UK).

#### 2.3.2. Transmission Electron Microscopy

Some square slices with a thickness of ~600 μm were electron-discharge machined from 298 K, 77 K and 4 K steels. The square slices were mechanically thinned to ~50 μm from both sides to avoid sample bending, which commonly causes curved extinction fringes. Subsequently, they were punched to obtain thin foils with a diameter of 3 mm using a gatan 659 puncher. These thin foils were further thinned using a twin-jet electrolytic polisher (Struers TenuPol-5, Struers Inc., Copenhagen, Denmark) to acquire thin regions, which surround a hole in the thin foil and are thin enough to cause the electron beam to penetrate. Mechanical twins, dislocation configurations and deformation bands were observed using an FEI Tecnai G^2^ F20 (FEI Co. Ltd., Hillsboro, OR, USA) field-emission transmission electron microscope (FE-TEM) operated at 200 kV.

## 3. Results and Discussion

### 3.1. Tensile Properties at Different Temperatures

**Figure 1:**
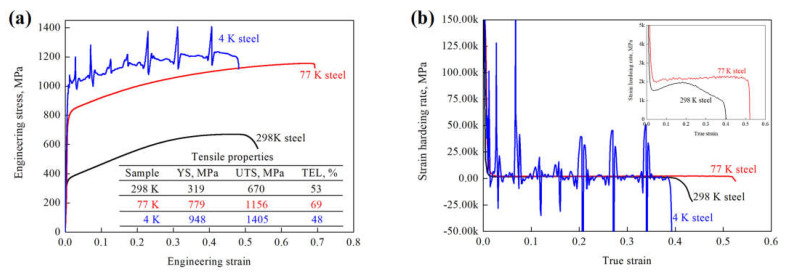
(**a**) Engineering stress–strain curves and (**b**) corresponding SHR curves derived from true stress–strain curves of the 298 K, 77 K and 4 K steels.

**Figure 2:**
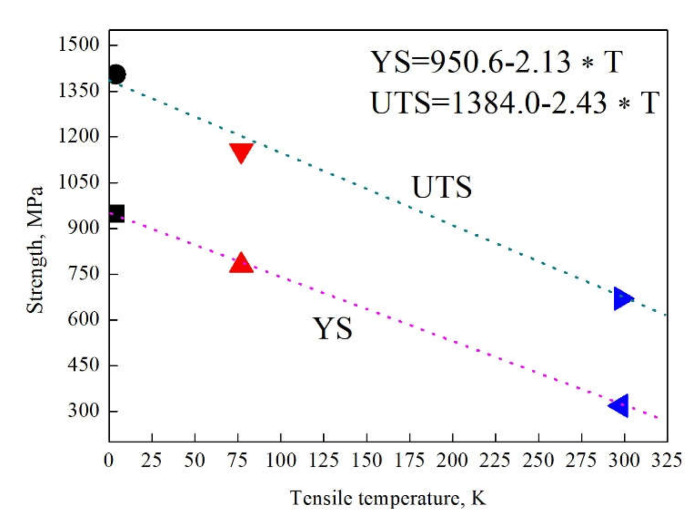
Correlation between strength and deformation temperature. The dark, red and blue colors represent the strengths of the steel deformed at 298, 77 and 4 K, respectively.

The uniaxial tensile engineering stress–strain and corresponding strain hardening rate (SHR) curves are shown in Figure 1. The yield strength (YS), ultimate tensile strength (UTS) and total elongation (TEL) are provided in the table and inserted in Figure 1a, showing that both YS and UTS can be greatly enhanced by lowering deformation temperature from 298 to 4 K, as a result of the high shear stress needed for dislocation gliding at a low temperature. The YS of the 77 K steel is ~2.4 times as high as that of the 298 K steel, and this value becomes ~3.0 as the deformation temperature further decreases to 4 K. Moreover, the correlation between YS and deformation temperature obeys a linear relationship, as shown in Figure 2. The UTS also shows a linear increase with the lowering of deformation temperature. The TEL increases from ~53 to ~69 % with the decreasing of deformation temperature from 298 to 77 K. Thus, the strength and ductility are simultaneously enhanced at a deformation temperature of 77 K. However, when the deformation temperature is further lowered to 4 K, there is a small and large decrease in TEL compared with the 298 K and 77 K steels, respectively. Note that the shape of the stress–strain curve of the 4 K steel is highly different from that of 298 K and 77 K steels. The stress–strain curves of the 298 K and 77 K steels are relatively smooth, whereas numerous serrations in the stress–strain curve of the 4 K steel can be clearly seen. In fact, this phenomenon was commonly observed in 316LN, 304L, medium-entropy alloy, etc. [18,19,20], and was interpreted by twinning, martensite transformation, the formation of burst dislocations or adiabatic deformation [19,20,21,22]. However, in the present work, the martensite transformation has not occurred at 4 K, indicating that it is not a reason for the formation of serrations.

In addition, the strain hardening rate was derived from the true stress–strain curves, as shown in Figure 1b, exhibiting a strong temperature dependence of SHR. In the early deformation stage, the SHRs sharply decrease for the three deformation temperatures as a result of dynamic recovery caused by cross slipping and annihilation of dislocations as well as the formation of low energy dislocation structures [13]. In the final deformation stage, the SHRs also sharply decrease because of the saturation of dislocation multiplication and the formation of denser and thicker twins [1]. However, in the main deformation stage, the SHR of the 298 K steel is nearly the same as typical high manganese TWIP steels, showing a slight increase owing to mechanical twinning and a further decrease because of less active mechanical twinning [1,23]. The 77 K steel possesses a higher SHR compared with the 298 K steel, and the SHR becomes nearly constant, appearing as if the mechanical twinning is sufficiently and continuously activated. Moreover, the dynamic recovery can be effectively suppressed at a low temperature, which also generates dislocation storage [24]. When the deformation temperature is lowered to 4 K, numerous peaks in the SHR curve can be observed. This result should not be caused by dynamic strain aging due to the very low diffusivity of carbon at 4 K and the addition of aluminum. In fact, the localized plastic instabilities lead to serrations, which are frequently observed in 316LN, 304L, medium-entropy alloy and Ti alloys, etc., at low temperatures because of the combination of thermal heat flow capacity and high flow stress [25].

### 3.2. Microstructure Characteristics before Deformation

**Figure 3:**
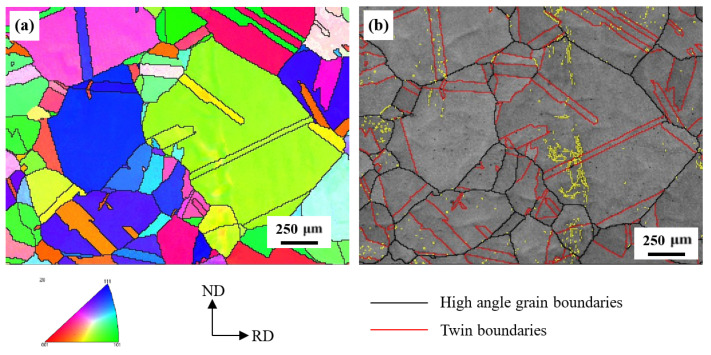
EBSD crystallographic analyses of the steel before deformation. (**a**) IPF map, showing grain orientations. (**b**) IQ map containing general high angle grain boundaries and Σ3 twin boundaries.

Figure 3 provides an EBSD-inverse pole figure (IPF) map as well as an image quality (IQ) map with general grain boundaries and twin boundaries. There is nearly no color contrast in the grain interior, implying very low local orientation gradients in the grain interior. Numerous annealing twins can be observed, indicating that annealing twins were not suppressed by the addition of ~5.0 wt% Al. Moreover, note that a lot of annealing twins go through the whole grain, whereas some Σ3 {112} incoherent twin boundaries terminate in the grain interior. It is commonly accepted that the twinning in face-centered-cubic crystal is through layer-by-layer displacement of a/6<112> Shockley partial dislocations on consecutive {111} planes [26,27,28]. Thus, these partial dislocations can be blocked by pre-existing dislocation tangles [29], resulting in the termination of annealing twins in the grain interior. In addition, the average grain size, considering annealing twins, was estimated to be 122 μm using a linear intercept method.

### 3.3. Microstructure Characteristics after Tensile Deformation at Different Temperatures

#### 3.3.1. EBSD Observations

**Figure 4:**
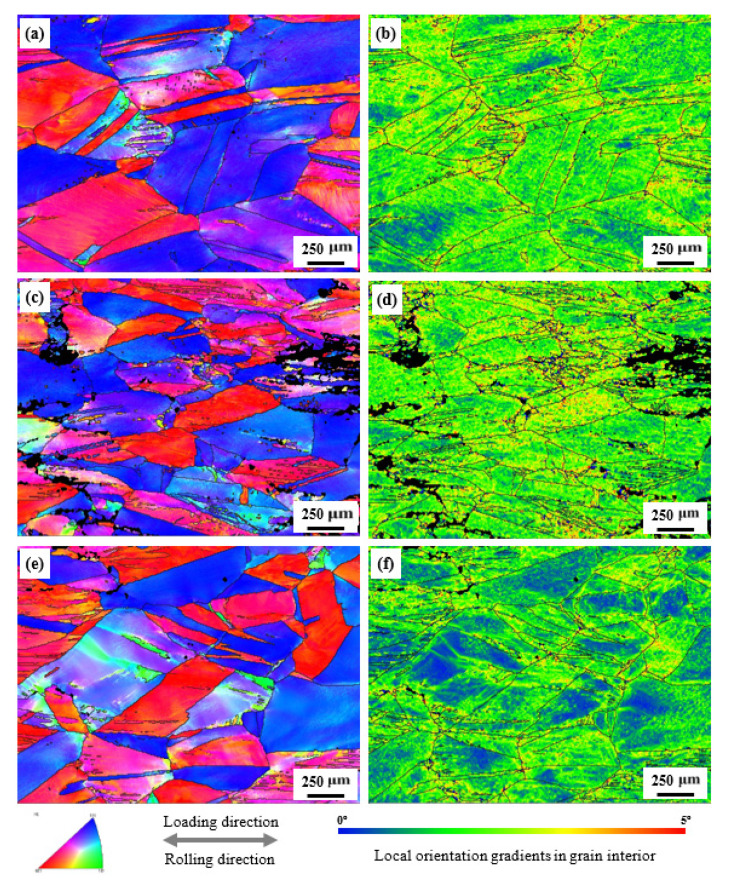
EBSD IPF and KAM maps of the steels subjected to a maximum uniform tensile deformation at (**a**,**b**) 298 K, (**c**,**d**) 77 K as well as (**e**,**f**) 4 K.

The EBSD IPF and kernel average misorientation (KAM) maps of the steels, subjected to a maximum uniform tensile deformation at 298, 77 and 4 K, are shown in Figure 4. Regardless of the deformation temperatures, almost all grains were prolonged along the rolling direction//loading direction, nearly all grains tended to align along the <001>//tensile direction or the <111>//tensile direction, and there existed an obvious color contrast in the grain interior. Figure 3 shows that there is nearly no preferred orientation of austenite grains in the steel before deformation. Thus, the observations of <001> or <111>//tensile direction indicates that the grains with orientations deviating from <001> or <111> reorientated during deformation. In addition, the pronounced color contrast in the grain interior always implies severe lattice distortion and plastic deformation, leading to a large local misorientation, which can be supported by KAM maps. Figure 4d,f show most regions with KAM values higher than 2°. A high KAM value always represents a high dislocation density, a wide distribution of misorientation and a high accumulation of plastic deformation [7]. Hence, a relatively large plastic deformation occurred in the 298 K, 77 K and 4 K steels. Note that there exist some regions with KAM values close to or greater than 4°, and these regions are commonly observed in the vicinity of grain boundaries, implying the piling up of more dislocations at the grain boundaries. Moreover, the area of the regions with KAM values close to or greater than 4° in the 77 K steel is higher than those in the 298 K and 4 K steels. In addition, there are some dark regions in the 77 K steel, indicating low confidence index values because of strong lattice distortion [30]. Furthermore, the areas of the regions with KAM values less than 1° in the 298 K and 4 K steels are greater than those in the 77 K steels. These results indicate that the steel possesses the best tensile plastic deformation capacity. Although the EBSD results clearly exhibit grain orientations, the degree of plastic deformation, as well as some twin segments and substructures, cannot be clearly observed due to a relatively low resolution of SEM. Hence, these substructures were observed by means of TEM.

#### 3.3.2. TEM Observations

**Figure 5:**
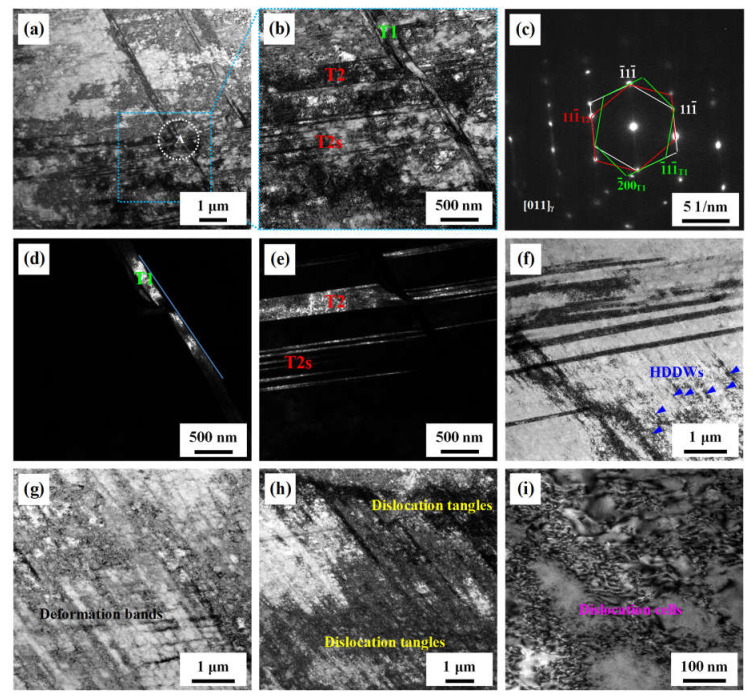
TEM images of typical microstructures of the steel after tensile deformation at 298 K. (**a**,**b**): Bright-field TEM images, exhibiting clear contrast of two-variant mechanical twins, and (**b**) is an enlarged image of the square outlined region in (**a**). (**c**) SAEDP of the outlined region A in (**a**). (**d**,**e**): Corresponding dark-field TEM images obtained from the ${\left(\bar{2}00\right)}_{T1}$ and ${\left(11\bar{1}\right)}_{T2}$ reflections in (**c**). (**f**–**i**): Bright-field TEM image, showing HDDWs, deformation bands, dislocation tangles and dislocation cells.

The representative microstructure characteristics of the steel subjected to a maximum uniform tensile deformation at 298 K are shown in Figure 5, exhibiting deformation mechanisms consisting of mechanical twinning, planar dislocation gliding as well as cross slipping and climbing of dislocations. Figure 5a,b show that the two-variant twinning systems of $\left(11\bar{1}\right)\left[\bar{2}1\bar{1}\right]$ and $\left(\bar{1}1\bar{1}\right)\left[\bar{2}\bar{1}1\right]$ were activated during tensile deformation at 298 K, providing evidence that a slight increase in the SHR curve results from mechanical twinning. In order to clearly show the morphology of two-variant twins, they are highlighted in Figure 5d,e. It can be clearly seen that the T1 twin becomes bent after twin–twin intersection, indicating strong twin–twin interactions, which can accommodate plastic deformation and enhance SHR [31]. Additionally, there are numerous T2 twins with fine spacing, leading to a sharp decrease in the mean free path of dislocation. Moreover, there exist contrasts in the dislocations between mechanical twins, indicating a sufficient dislocation storage. Thus, the SHR can be increased or kept at a high value. Although the SFE of the steel is as high as ~63.6 mJ/m^2^ at 298 K, numerous mechanical twins can be observed in the 298 K steel, and the SHR curve also shows that the mechanical twinning has occurred. In general, the mechanical twinning can be activated for the SFE ranging from 15~20 to 40~50 mJ/m^2^ [11,32,33,34], implying that a SFE of ~63.6 mJ/m^2^ exceeds the upper limit of the SFE range for mechanical twinning. The reason for the activation of mechanical twinning in the 298 K steel is large grain size, which can sufficiently lower twinning stress [35].

Besides mechanical twinning, Figure 5f exhibits highly dense dislocation walls (HDDWs) along one variant {111} slip plane, and this dislocation configuration is always formed by dense dislocation sheets lying on the primary slip system [36], implying that the planar dislocation glide should be another deformation mechanism. In addition, the deformation bands, dislocation tangles and dislocation cells can be also observed. These substructures also provide some contributions to SHR.

**Figure 6:**
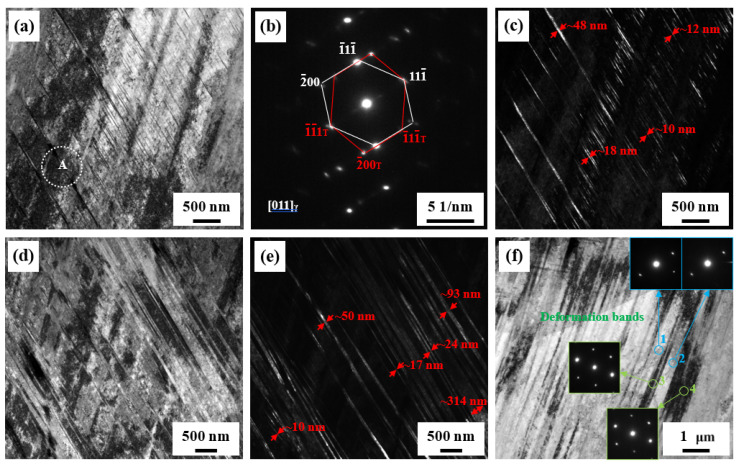
TEM images of typical microstructures of the steel after tensile deformation at 77 K. (**a**,**d**): Bright-field TEM images, exhibiting clear contrast of mechanical twins. (**b**) SAEDP of the outlined region A in (**a**). (**c**,**e**): Corresponding dark-field TEM images obtained from the ${\left(\bar{1}1\bar{1}\right)}_{T}$ reflection in (**b**). (**f**) Bright-field TEM image, showing deformation bands. The insets in (**f**) are SAEDPs of the outlined regions 1–4 in (**f**).

When the deformation temperature is decreased to 77 K, the deformed microstructure is very different from the 298 K steel, as shown in Figure 6. Although the SFE of the steel is as high as ~63.6 mJ/m^2^ at 298 K, this value decreases to ~38.6 mJ/m^2^ at 77 K. Thus, the mechanical twinning can be sufficiently activated according to the SFE range for mechanical twinning. Numerous mechanical twins are indeed observed in the 77 K steel. Most mechanical twins show a relatively small thickness of 10~20 nm or large thickness of 50~100 nm, and there are some thicken mechanical twins with a thickness greater than 300 nm, as shown in Figure 6e. Compared with the 298 K steel, there are two major differences in the morphology of mechanical twins. One is that the volume fraction of mechanical twins is far higher than that in the 298 K steel. The other one is that the distribution of mechanical twins is very dense, leading to small twin spacing. Large volume fraction of mechanical twins and very small twin spacing should provide a major contribution to high SHR. Thus, the SHR of the 77 K steel is higher than that of the 298 K steel and remains nearly constant during the main deformation stage, supporting the fact that the mechanical twinning indeed enhances SHR and ductility. Hence, the TEL of the 77 K steel is higher than that of the 298 K steel. Some deformation bands can be also observed, as shown in Figure 6f. The regions with bright contrast have almost the same orientation, and the regions with dark contrast also have similar orientations. Moreover, there is a small deviation in orientation between the two contrasted regions. These deformation bands contribute to SHR to varying degrees [36].

**Figure 7:**
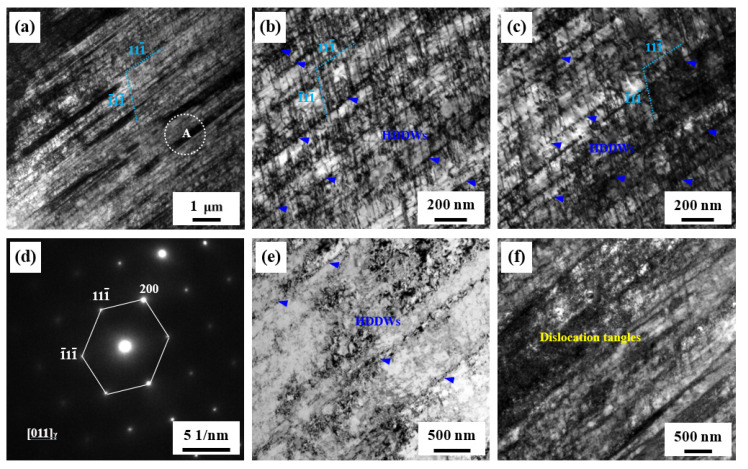
TEM images of typical microstructures of the steel after tensile deformation at 4 K. (**a**–**c**): Bright-field TEM images, showing HDDWs and planar dislocation slipping. (**d**) SAEDP of the outlined region A in (**a**). (**e**,**f**): Bright-field TEM images, showing HDDWs and dislocation tangles.

Figure 7 provides typical microstructures of the steel after tensile deformation at 4 K. Figure 7a shows numerous dense bands with a dark contrast along the $\left(11\bar{1}\right)$ planes, and numerous dislocations can be also observed between bands. In order to show the clear morphology of these bands, some regions were magnified and shown in Figure 7b,c. Numerous slip traces along the $\left(\bar{1}1\bar{1}\right)$ planes can be also observed. Thus, the formation of these dislocation configurations is due to dislocations pile up on the $\left(11\bar{1}\right)$ and $\left(\bar{1}1\bar{1}\right)$ planes [37,38], leading to the formation of dense dislocation networks of planar dislocation arrays forming the so-called Taylor lattices [36]. Figure 7e shows that some regions contain HDDWs along the primary slip planes. In addition to the above-mentioned dislocation configurations, the dislocation tangles can be also observed, indicating a high dislocation accumulation and strong dislocation interactions. Moreover, it can be seen that the dislocation density between ill-defined boundaries is always high. These bands are also termed microbands [37,38].

With the further lowering of the deformation temperature to 4 K, few mechanical twins can be observed. Instead, the dislocation configurations are characterized by planar dislocation arrays. Although the SFE of the steel is ~34.4 mJ/m^2^ at 4 K and this value is slightly less than ~38.6 mJ/m^2^, a lot of mechanical twins, such as those in the 77 K steel, are not observed, seeming to indicate that the mechanical twinning is hard to activate at 4 K. Compared with the 77 K steel, a pronounced decrease in TEL may be due to few mechanical twins. However, compared with the 298 K steel, there is only a small decrease in TEL, indicating that the formation of planar dislocation arrays and microbands can also enhance SHR, and a relatively high TEL can be obtained. In addition, the serrations in the stress–strain curves of metals tested at 4 K are commonly observed, and some mechanisms were proposed to explain this phenomenon. We think that the adiabatic deformation is the main reason. Furthermore, the two-variant planar dislocation arrays in the 4 K steel may be also a reason for the formation of serrations. The pre-formed planar dislocation arrays can act strong barriers for dislocation slipping along another variant slip plane, and dislocation pinning and depinning can occur, generating bursts of increased and decreased stress.

## 4. Conclusions

In the present work, the tensile deformation behaviors of a high manganese austenitic steel were investigated at 298, 77 and 4 K. The deformed microstructures were characterized by means of EBSD and TEM. Some conclusions can be drawn.

1. It is normal that both YS and UTS increased with the lowering of deformation temperature, whereas the TEL exhibited an obvious increase at 77 K and only a slight decrease in TEL at 4 K compared with the one at 298 K, thus overcoming the long-standing strength–ductility trade-off.
2. In the main deformation stage, the SHR of the 298 K steel was typical for high manganese TWIP steels. A high and constant SHR led to a high ductility in the 77 K steel. However, the 4 K steel exhibited multi-peak strain hardening behaviors.
3. With the lowering of deformation temperature, the major change in the deformation mechanism was as follows: certain twinning → sufficient twinning → little twinning. This change is consistent with the change in TEL, indicating that the mechanical twinning indeed provided a high and stable strain hardening capacity. Thus, a high TEL could be obtained.
4. Interestingly, it was found that although the SFE of the steel was ~34.4 mJ/m^2^ at 4 K, sufficient twinning was not observed. Instead, numerous planar dislocation arrays and microbands could be observed. This may be also a reason for the formation of serrations.

## Figures and Tables

**Table 1:** Chemical compositions and SFEs of the steel, wt%.

C	Si	Mn	Al	P	S	Fe	SFE (298 K), mJ/m^2^	SFE (77 K), mJ/m^2^	SFE (4 K), mJ/m^2^
0.6	0.5	24.2	4.9	0.0029	0.011	balance	63.6	38.6	34.4

## Data Availability

The raw/processed data required to reproduce these findings cannot be shared at this time as the data also forms part of another ongoing study.
